# Breast lump, a rare presentation of costochondral junction tuberculosis: a case report

**DOI:** 10.4076/1757-1626-2-7039

**Published:** 2009-09-09

**Authors:** Sanjay Jain, Adesh Shrivastava, Dinesh Chandra

**Affiliations:** Department of General Surgery, Gandhi Medical College and Associated HospitalsBhopal-462001, Madhya PradeshIndia

## Abstract

The diagnosis of musculoskeletal tuberculosis remains a challenge for clinicians and requires a high index of suspicion. The combination of indolent onset of symptom and signs with histological or cytological features and compatible radiography findings, strongly suggest the diagnosis. Prompt diagnosis and treatment are important to prevent serious bone and joint destruction. Tuberculosis involving ribs and presenting with breast mass is a very rare entity and only a few cases have been reported in the literature previously. We present a case of 30-year-old female having tuberculous involvement of the costochondral junction and presenting as a lump in the lower inner & outer quadrant of right breast. Surgical exploration and histopathological evaluation revealed costochondral junction tuberculosis and secondary abscess formation in the right breast. The lump was located in the breast rather than the usual retromammary location, when arising from chest wall or internal mammary nodes. This unusual manifestation of tuberculosis should be included in differential diagnosis of patients presenting with a breast mass and high risk of tuberculosis.

## Introduction

The thoracic wall is an uncommon location for tuberculosis, accounting for an estimated 1-5% of all the cases of musculo-skeletal tuberculosis which themselves account for 15% of all extrapulmonary localization [1]. Because of the rarity of tuberculosis as a cause of symptomatic breast disease, the diagnostic efforts were directed at the more common causes such as a carcinoma and other benign lesions and the diagnosis of tuberculosis has often been missed [2]. However, unlike other conditions, TB is eminently curable and therefore it is imperative that clinicians should bear it in mind when managing patients with breast disease symptoms. We present an interesting case of costochondral junction TB in this report.

## Case presentation

A 30-year-old Indian female patient of Asian ethnicity presented with lump in lower half of right breast of 12 × 7 cm size of two month duration. It had begun as a small parasternal swelling which had enlarged to involve the inner and outer lower quadrants of right breast. The lump was smooth, lobulated, soft to firm and non tender. The overlying skin had erythema and superficial skin erosion but with no rise of skin temperature, pain or constitutional symptoms of inflammation. The patient had no history or clinical features of pulmonary Koch’s. Chest X-ray was unremarkable. A high frequency ultrasound demonstrated an irregular mass with solid and cystic consistency in the lower inner & outer breast quadrants with the deeper extent dipping into the chest wall. Fine needle aspirate was positive for acid fast bacilli and a diagnosis of chest wall tuberculosis with secondary breast involvement was made. Excision was planned under cover of anti tubercular drugs, labelling the lump to arise from one of the internal mammary nodes and secondarily involving the breast. It was excised using an elliptical incision and exploration for inner extent ended in a punched out lesion at the fifth costochondral junction. No trans-muscular track was found and hence primary costochondral junctional origin was confirmed. Histopathological examination of the specimen showed caseating granulomas with epitheloid histiocytes and Langerhan’s giant cells. Recovery was uneventful and the patient was discharged from the hospital on 3^rd^ post operative day with anti tubercular treatment to be continued.

## Discussion

When involving the bone, tuberculosis (TB) is thought to result from either lymphatic or haematogenous dissemination of bacilli from a site of primary infection, usually a Ghon’s focus, in the lung. TB of the chest wall constitutes 1% to 5% of all cases of musculoskeletal TB [1,3-6], and can involve the sternum, costochondral junctions, rib shafts, costovertebral joints and the vertebrae [7]. Faure et al [8] hypothesized that infection of lymph nodes in the chest, which also includes the internal mammary group, result secondary to pulmonary kochs. These then caseate and can erode through the chest wall. They usually track through intercostals muscles to form a visible swelling on the exterior without erythema or tenderness. The costochondral & costovertebral junctions, and vertebrae are involved less frequently [9].

Tuberculosis of breast is a relatively rare condition when compared with frequency of tuberculous infection in other organs of human body, yet it is not so uncommon among the disease of the breast. (Morgen 1931) The portal of entry can be hematogenous, lymphatic or by a caseating internal mammary node, abscess being usually retro-mammary in the latter. In developing countries where tuberculosis is common the diagnosis can be made easily by evaluating clinical course and signs, but investigations are necessary to confirm the diagnosis [10].

A plain chest radiograph is taken as part of the routine work for patient with breast mass. The detection of apical lung lesion that is suggested present or past tuberculosis can provide diagnostic clues in the differential diagnosis of suspected lesion. The presence of tuberculosis lesions in the lungs may suggest the diagnosis. However it should be kept in mind that a small percentage of the tuberculosis cases could be seen without lung involvement.

Mammography and USG are the primary imaging methods in the evaluation of breast lesions. But the mass lesions near the chest wall cannot be evaluated enough due to technique difficulties in positioning the patient. Computerised tomography has the advantage of demonstrating deep and superficial tissues in same contrast and spatial resolution with additional rim enhancement on contrast, thus providing most diagnostic information [11]. Definitive treatment involves use of anti tubercular drugs and surgical intervention if indicated.

This case underlines the important message that tuberculosis should be considered when breast mass is undiagnosed, especially in at-risk populations.

## Conclusion

The granulomatous inflammation of tuberculosis usually involves the lungs and the hilar lymph nodes, and anterior chest wall involvement is very rare. When extending out of the chest, the position is usually retromammary. The diagnosis of musculoskeletal tuberculous infection remains a challenge for clinicians and requires a high index of suspicion. The combination of Clinical course and finding with supporting laboratory investigation strongly suggests the diagnosis. TB treatment is often started immediately after the appropriate microbiological and histological samples have been obtained if the clinical suspicion is high. But at the same time it must be confirmed by positive culture or histopathological examination. Prompt diagnosis and treatment are important to prevent serious bone and joint destruction and future clinical course.

**Figure 1.**
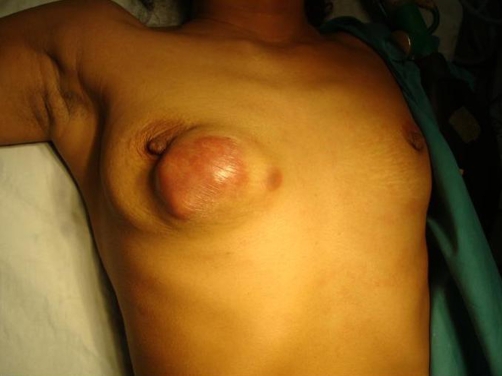
Pre operative photograph showing the lump in right breast.

**Figure 2.**
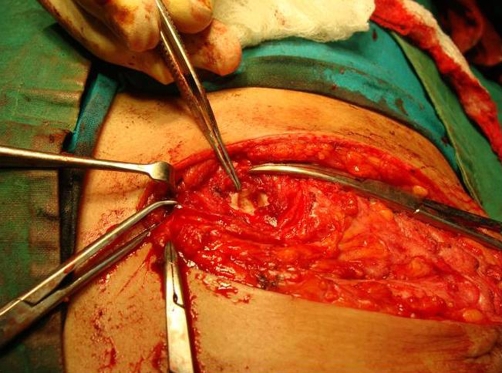
Intra operative photograph showing a punched out lesion at the right 5^th^ costochondral junction.
